# A retrospective study on influencing factors of postoperative hospital stay and development of a predictive scoring model for elderly patients (≥70 years) with colorectal cancer undergoing laparoscopic radical resection

**DOI:** 10.3389/fsurg.2025.1709437

**Published:** 2025-11-28

**Authors:** Jinyi Zhou, Dongyang He, Hui Yao, Zhengfei Zhao

**Affiliations:** 1 Department of Gastrointestinal Surgery, The Affiliated Hospital of Southwest Medical University, Luzhou, China; 2 Department of General Surgery, Chengdu Fifth People’s Hospital, Chengdu, China

**Keywords:** elderly colorectal cancer, laparoscopic radical resection, postoperative hospital stay, predictive model, Cox regression analysis

## Abstract

**Background:**

With the accelerating global population aging, the proportion of elderly patients with colorectal cancer (CRC) undergoing laparoscopic radical resection is increasing annually. However, significant individual variations in postoperative hospital stay exist, and convenient clinical prediction tools remain lacking. This study aimed to develop and validate a simplified predictive scoring model for postoperative hospital stay in elderly CRC patients after laparoscopic radical resection.

**Materials and methods:**

A total of 205 elderly CRC patients (≥70 years) who underwent laparoscopic radical resection at our hospital from August 2024 to September 2025 were retrospectively included. Baseline characteristics (age, sex, BMI, comorbidities), tumor indicators (location, TNM stage), surgical parameters (operative time, blood loss, stoma creation), preoperative and postoperative blood markers (albumin, hemoglobin, direct bilirubin), perioperative assessments (ASA classification), and postoperative outcomes (30-day complications, hospital stay) were collected. Postoperative hospital stay (excluding delays due to non-medical factors) served as the primary outcome. Univariate linear regression identified potential influencing factors, and multivariate linear regression determined independent risk factors. A predictive scoring model was constructed based on independent factors, with efficacy validated using the coefficient of determination (*R*^2^) and root mean square error (RMSE). Risk stratification was performed to analyze differences in hospital stay across scoring tiers.

**Results:**

The mean postoperative hospital stay was 16.6 ± 5.0 days (range: 9–42 days). multivariate linear regression analysis revealed that 30-day postoperative complications were an independent risk factor for prolonged hospital stay (*β* = 7.689, *P* < 0.001). A simplified scoring model was developed: postoperative complications (present = 3 points, absent = 0 points), ≥2 comorbidities (yes = 1 point, no = 0 points), and operative time >180 min (yes = 1 point, no = 0 points), yielding a total score range of 0–5 points. Risk stratification showed: low-risk group (0 points) had an expected stay of 14.8 ± 3.2 days (15% of patients), medium-risk group (1–2 points) 16.0 ± 4.0 days (60%), and high-risk group (3–5 points) 22.5 ± 6.8 days (25%).

**Conclusion:**

The simplified scoring model developed in this study effectively predicts postoperative hospital stay in elderly CRC patients undergoing laparoscopic radical resection, providing a practical tool for clinical risk stratification, early intervention, and optimization of healthcare resources.

## 1 Introduction

Colorectal cancer (CRC) represents a major global health burden, with its incidence and mortality disproportionately affecting elderly populations. According to GLOBOCAN 2020 statistics, individuals aged ≥70 years account for 42%–48% of all new CRC diagnoses worldwide, and this proportion is projected to rise by 25% by 2035 due to global population aging (1). In China, national cancer registry data further highlight this trend: CRC incidence in adults ≥70 years has increased by 11.3% annually over the past decade, making it the second most common malignancy in this age group and a leading cause of cancer-related death (2).

Beyond chronological aging, the rising CRC burden is also driven by evolving dietary habits—characterized by increased intake of high-calorie, high-fat foods and the gradual abandonment of the Mediterranean diet (rich in vegetables). Such dietary shifts alter the intestinal microbiota, and emerging evidence indicates that interactions between the gut microbiome and mucosal cellular genes contribute to the increasing incidence of CRC. While this is not the sole etiological factor, recent studies have underscored its clinical importance, providing a more comprehensive understanding of CRC pathogenesis (3).

For elderly CRC patients, curative-intent surgical resection remains the cornerstone of treatment, and laparoscopic radical resection has emerged as the standard of care in tertiary medical centers. Compared with open surgery, laparoscopy offers well-documented advantages for elderly patients, including reduced intraoperative blood loss, lower postoperative pain scores, faster gastrointestinal function recovery, and decreased overall complication rates (4, 5). However, a critical clinical challenge persists: even among patients undergoing laparoscopic radical resection, postoperative hospital stay (POHS) varies dramatically—from 9 to 42 days in our preliminary cohort. This variability not only exacerbates bed resource shortages in high-volume centers but also increases the risk of nosocomial infections, delirium, and functional decline in elderly patients with limited physiological reserve (6, 7).

Existing research on POHS in elderly CRC patients has three key limitations. First, most studies focus on “laparoscopic vs. open surgery” comparisons rather than investigating factors specific to the laparoscopic cohort (8, 9). With laparoscopy now accounting for >80% of CRC resections in elderly patients at tertiary centers, there is an urgent need to understand drivers of POHS within this homogeneous surgical group. Second, previous studies often rely on complex or non-routinely collected variables (e.g., preoperative CEA levels, lymph node yield, molecular subtypes) to predict POHS, making their models impractical for bedside application in busy clinical settings (8, 10). Third, few studies quantify the incremental impact of modifiable factors (e.g., preoperative nutritional optimization, intraoperative surgical duration control) on POHS, limiting their ability to guide preventive interventions (11). And most critically, current studies rarely integrate functional, frailty, or physiological reserve measures—core domains of geriatric surgery. In recent years, frailty has overtaken chronological age as the most robust predictor of adverse postoperative outcomes in elderly patients: a study demonstrated that frail patients (identified via the Clinical Frailty Scale) have a 2.8-fold higher risk of prolonged POHS than non-frail peers, even after adjusting for comorbidity count (12). Similarly, handgrip strength—a simple bedside frailty marker—was shown to predict POHS more strongly than comorbidities alone in abdominal surgery patients, with each 5 kg decrease in grip strength associated with a 1.2-day extension of hospital stay (13). This gap means existing models fail to capture the “biological age” of elderly patients, limiting their applicability to geriatric surgical practice.

Against this backdrop, we conducted a large single-center retrospective study of 205 elderly CRC patients (≥70 years) who underwent laparoscopic radical resection. Our specific objectives were: (1) to identify independent clinical factors influencing POHS using routinely collected data (e.g., comorbidities, surgical metrics, postoperative complications); (2) to develop a simplified predictive scoring model based on these factors, prioritizing ease of use and data accessibility; and (3) to validate the model's efficacy for risk stratification, thereby providing a practical tool to optimize perioperative management and reduce unnecessary POHS in this vulnerable population.

## 2 Materials and methods

### 2.1 Study population

#### 2.1.1 Inclusion criteria

1. Age ≥70 years;
2. Postoperative pathological confirmation of primary colorectal adenocarcinoma;
3. Undergoing laparoscopic radical resection (R0 resection, including laparoscopic-assisted and totally laparoscopic procedures; exclusion of robot-assisted and conversion to open surgery);
4. Complete clinical data (including baseline characteristics, surgical parameters, hematological indicators, and complication records);
5. Discharge due to “improved/cured condition” (excluding non-medical factors such as family-requested delayed discharge or transfer to other departments).

#### 2.1.2 Exclusion criteria

1. Preoperative distant metastasis (TNM stage IV) or emergency surgery (e.g., intestinal obstruction, massive hemorrhage);
2. Palliative resection or local tumor excision;
3. Concomitant severe organ failure (e.g., end-stage chronic heart failure, uremia);
4. Abnormally prolonged hospitalization due to other diseases (e.g., acute myocardial infarction) during admission;
5. Conversion to open surgery during laparoscopy.

A total of 78 patients were excluded.

#### 2.1.3 Final cohort

205 patients admitted to our hospital from August 2024 to September 2025 were enrolled.

### 2.2 Data collection

All data were extracted from inpatient medical records (no follow-up required) and organized by “clinical logic dimensions” to ensure comprehensive coverage of all collected indicators (Table 1):

**Table 1:** Clinical data collection indicators, data sources, and classification/calculation methods for elderly patients with colorectal cancer (CRC) undergoing laparoscopic radical resection.

Data category	Specific indicators	Data source	Classification/calculation method
1. Baseline characteristics	Age (years), Gender (Male/Female), BMI (kg/m^2^)	Medical record front page, Physical examination	Age stratification: <80 years/≥80 years; BMI stratification: <18.5 (underweight)/18.5–25 (normal)/≥25 (overweight/obese)
2. Comorbidities	Hypertension, Diabetes, Cerebral infarction (preoperatively diagnosed), Coronary heart disease, COPD	Past medical history, Discharge diagnosis	“Number of comorbidities”: 0/1/≥2 (due to cumulative impact of multiple comorbidities on recovery)
3. Tumor-related	Tumor location (Left colon/Right colon/Rectum), TNM stage (AJCC 8th edition)	Preoperative imaging, Postoperative pathology	TNM stratification: Stage I–II/Stage III (Stage IV excluded)
4. Perioperative assessment	ASA classification (American Society of Anesthesiologists)	Anesthesia record, Preoperative evaluation summary	Stratification: ASA I–II (low-risk)/ASA III (high-risk) (ASA IV patients excluded)
5. Laparoscopic surgical indicators	Operative time (min, skin incision to closure), Estimated blood loss (ml, anesthesia record), Intraoperative stoma creation (None/Temporary/Permanent)	Surgical record, Anesthesia record	Operative time stratification: ≤180 min/>180 min; Stoma focus: “Temporary stoma” (permanent stomas merged into “stoma present” due to low incidence)
6. Hematological indicators	Preoperative (within 1 week): Albumin (Alb), Hemoglobin (Hb), Direct bilirubin (DBIL); Postoperative day 1: Alb, Hb	Preoperative labs, Postoperative day 1 labs	Preoperative Alb: <35 g/L (hypoalbuminemia)/≥35 g/L; Preoperative Hb: <100 g/L (anemia)/≥100 g/L; Preoperative DBIL: >6 μmol/L (abnormal)/≤6 μmol/L (normal)
7. Postoperative outcomes	30-day postoperative complications (intestinal obstruction, bleeding, infection requiring intervention), Postoperative hospital stay	Nursing records, Postoperative progress notes, Discharge summary	Complication classification: Single complication (e.g., infection only)/Multiple complications (e.g., infection + obstruction); Hospital stay: Discharge date—Surgery date (calendar days)

### 2.3 Statistical methods

Data were analyzed using SPSS 26.0 and visualized with GraphPad Prism 9.0.

#### 2.3.1 Descriptive statistics

Continuous variables (operative time, blood loss, hospital stay) were presented as mean ± standard deviation (*x¯* ± *s*), supplemented by median (interquartile range). Categorical variables (comorbidities, complications) were expressed as *n* (%).

#### 2.3.2 Risk factor screening

Univariate linear regression: Baseline characteristics, comorbidities, tumor indicators, surgical parameters, and hematological indicators were included as independent variables, with postoperative hospital stay as the dependent variable. Variables with *P* < 0.1 were considered potential influencing factors.

Multivariate linear regression: A stepwise regression method (entry *α* = 0.05, removal *α* = 0.1) was applied to identify independent risk factors after controlling for confounders (e.g., gender, BMI).

For variables with marginal significance (0.05 < *P* < 0.1) in multivariate analysis (i.e., comorbidity count ≥2 types, operative time >180 min), we retained them in the final model based on two criteria: (1) clinical plausibility—previous studies have confirmed that multimorbidity (≥2 comorbidities) increases physiological stress in elderly patients, prolonging recovery by 1.0–1.5 days (12), and operative time >180 min is associated with higher intraoperative inflammation, which delays gastrointestinal function recovery (13); (2) model practicality—these variables are routinely recorded in electronic medical records, ensuring the model's bedside applicability.

#### 2.3.3 Development and validation of the scoring model

##### 2.3.3.1 Assignment logic

Scores were assigned based on the absolute values of standardized regression coefficients (*β*) derived from multivariate regression analysis, generating a total score ranging from 0 to 5 points.

##### 2.3.3.2 Risk stratification

Patients were stratified into three risk tiers according to the total score.

##### 2.3.3.3 Model validation

Explanatory Power: The coefficient of determination (*R*^2^) was used to evaluate the model's ability to explain variability in postoperative hospital stay.

Prediction Accuracy: The root mean square error (RMSE) was calculated to quantify prediction error, with lower RMSE values indicating higher precision.

## 3 Results

### 3.1 Baseline characteristics and laparoscopic surgical details

The baseline characteristics and surgical details of 205 elderly patients who underwent laparoscopic radical resection for colorectal cancer are presented in Table 2. Among the 205 patients, the mean age was 75.7 ± 4.9 years (range: 70–98 years), with 40 patients (19.5%) aged ≥80 years. There were 119 males (58.0%). The mean BMI was 22.1 ± 3.1 kg/m^2^, including 21 underweight patients (10.2%), 148 normal-weight patients (72.2%), and 36 overweight/obese patients (17.6%). Comorbidities included hypertension (*n* = 91, 44.4%), diabetes (*n* = 42, 20.5%), cerebral infarction (*n* = 25, 12.2%), coronary heart disease (*n* = 38, 18.5%), and COPD (*n* = 58, 28.3%). Overall, 73 patients (35.6%) had ≥2 comorbidities. Tumor location: Left colon (*n* = 22, 10.7%), right colon (*n* = 48, 23.4%), rectum (*n* = 135, 65.9%). TNM stage: Stage I–II (*n* = 135, 65.9%), Stage III (*n* = 70, 34.1%). ASA classification: ASA III (*n* = 73, 35.6%). Laparoscopic metrics: Mean operative time 237.9 ± 66.5 min (153 cases >180 min, 74.6%); mean blood loss 38.1 ± 33.6 mL. Intraoperative procedures included temporary stoma (*n* = 51, 24.9%) and permanent stoma (*n* = 17, 8.3%; permanent stoma cases were combined into the “stoma formation” group). Preoperative abnormalities: Hypoalbuminemia (Alb < 35 g/L; *n* = 39, 19.0%), anemia (Hb < 100 g/L; *n* = 51, 24.9%), elevated DBIL (>6 μmol/L; *n* = 24, 12.0%). Postoperative day 1: Alb decrease ≥5 g/L (*n* = 36, 18.0%).30-day complications (*n* = 49, 23.9%): Ileus (*n* = 17, 8.3%), bleeding (*n* = 12, 5.9%), infection (*n* = 20, 9.8%), and anastomotic leakage (*n* = 5, 2.4%); 7 cases (3.4%) had multiple complications. Notably, recent studies have emphasized that a healthy intestinal bacterial flora plays a protective role in reducing anastomotic leakage after colorectal surgery, which aligns with our observation of a relatively low anastomotic leakage rate (2.4%)—potentially attributed to unmeasured factors such as preoperative dietary patterns or gut microbiome status in our cohort (14).

**Table 2:** Baseline characteristics and surgical details of 205 elderly patients undergoing laparoscopic radical resection for colorectal cancer [*n* (%) or mean ± SD].

Variable	Value
Age (years)	75.7 ± 4.9
Age ≥ 80 years	40 (19.5)
Male	119 (58.0)
BMI (kg/m^2^)	22.1 ± 3.1
Underweight (<18.5)	21 (10.2)
Normal (18.5–25)	148 (72.2)
Overweight/obese (≥25)	36 (17.6)
Number of comorbidities	
0	51 (24.9)
1	81 (39.5)
≥2	73 (35.6)
ASA III	73 (35.6)
Tumor location	
Left colon	22 (10.7)
Right colon	48 (23.4)
Rectum	135 (65.9)
TNM stage III	70 (34.1)
Operative time >180 min	153 (74.6)
Blood loss (mL)	38.1 ± 33.6
Intraoperative stoma	51 (24.9)
Preoperative Alb < 35 g/L	39 (19.0)
Preoperative Hb < 100 g/L	51 (24.9)
Preoperative DBIL >6 μmol/L	24 (12.0)
30-day complications	49 (23.9)
Postoperative stay (days)	16.6 ± 5.0 (median 16)

### 3.2 Univariate and multivariate linear regression analyses of postoperative hospital stay

In the univariate Cox regression analysis for postoperative outcomes (prolonged hospital stay and complication occurrence) in elderly patients with colorectal cancer (CRC) who underwent laparoscopic radical resection, factors including age, physical status (assessed by ASA physical status classification), tumor characteristics, surgical process, and comorbidities were evaluated. The results showed that advanced age, higher ASA classification, advanced-stage tumor, large intraoperative blood loss, prolonged operation time, presence of comorbidities, and postoperative complications themselves were all independent risk factors for prolonged postoperative hospital stay and complication occurrence in these elderly CRC patients, with statistical significance (all *P* < 0.05) (Table 3).

**Table 3:** Univariate linear regression analysis of postoperative hospital stay.

Variable	Grouping	Prolonged hospital stay		Postoperative complications	
		HR [95% confidence interval (`CI)]	*P* value	HR (95% CI)	*P* value
Age group (Reference: 70–74 years)	75–79 years	1.18 (0.54–2.58)	0.686	1.45 (0.60–3.50)	0.407
	80–84 years	2.34 (1.12–4.89)	0.023	2.89 (1.24–6.74)	0.014
	≥85 years	7.58 (2.58–22.34)	<0.001	8.37 (2.42–28.94)	0.001
ASA physical status classification	Grade II	1.42 (0.65–3.12)	0.384	1.78 (0.72–4.41)	0.207
	Grade III	3.25 (1.28–8.26)	0.013	4.15 (1.56–11.03)	0.005
TNM stage	Stage II	1.89 (0.91–3.92)	0.089	2.34 (0.98–5.58)	0.055
	Stage III	4.32 (1.95–9.58)	<0.001	5.67 (2.12–15.17)	<0.001
Intraoperative blood loss	>200 mL	2.15 (1.33–3.47)	0.002	2.87 (1.65–4.99)	<0.001
Operation time	>180 min	1.78 (1.15–2.75)	0.010	2.12 (1.28–3.51)	0.004
Comorbidity	Diabetes mellitus	2.12 (1.28–3.51)	0.004	2.78 (1.58–4.89)	<0.001
	Chronic obstructive pulmonary disease (COPD)	2.84 (1.45–5.57)	0.002	3.45 (1.67–7.12)	0.001
Postoperative complications	Yes	3.42 (2.15–5.44)	<0.001	4.68 (1.08–20.26)	0.039

CRC, colorectal cancer; HR, hazard ratio; CI, confidence interval.

In the multivariate Cox regression analysis, however, only 30-day postoperative complications were identified as an independent risk factor for postoperative outcomes (e.g., prolonged hospital stay or complication occurrence) in elderly patients undergoing laparoscopic radical resection for CRC, with a high degree of statistical significance (*P* < 0.001). After adjusting for confounding effects, other factors such as age ≥80 years, presence of ≥2 comorbidities, preoperative albumin (Alb) level < 35 g/L, operation time >180 min, and intraoperative temporary enterostomy showed no statistically significant differences (all *P* > 0.05) (Table 4).

**Table 4:** Results of multivariate regression analysis for postoperative hospital stay.

Factor	*β* [standard error (SE)]	*P* value
Age ≥ 80 years	0.528 (0.692)	0.451
Comorbidity count ≥ 2 types	1.172 (0.698)	0.095
Preoperative albumin (Alb) < 35 g/L	0.143 (0.701)	0.836
Operation time >180 min	1.103 (0.632)	0.083
Intraoperative temporary enterostomy	0.234 (0.765)	0.756
30-day postoperative complications	7.689 (1.852)	<0.001

SE stands for standard error. *P* < 0.05 indicates a statistically significant difference.

**Figure 1:**
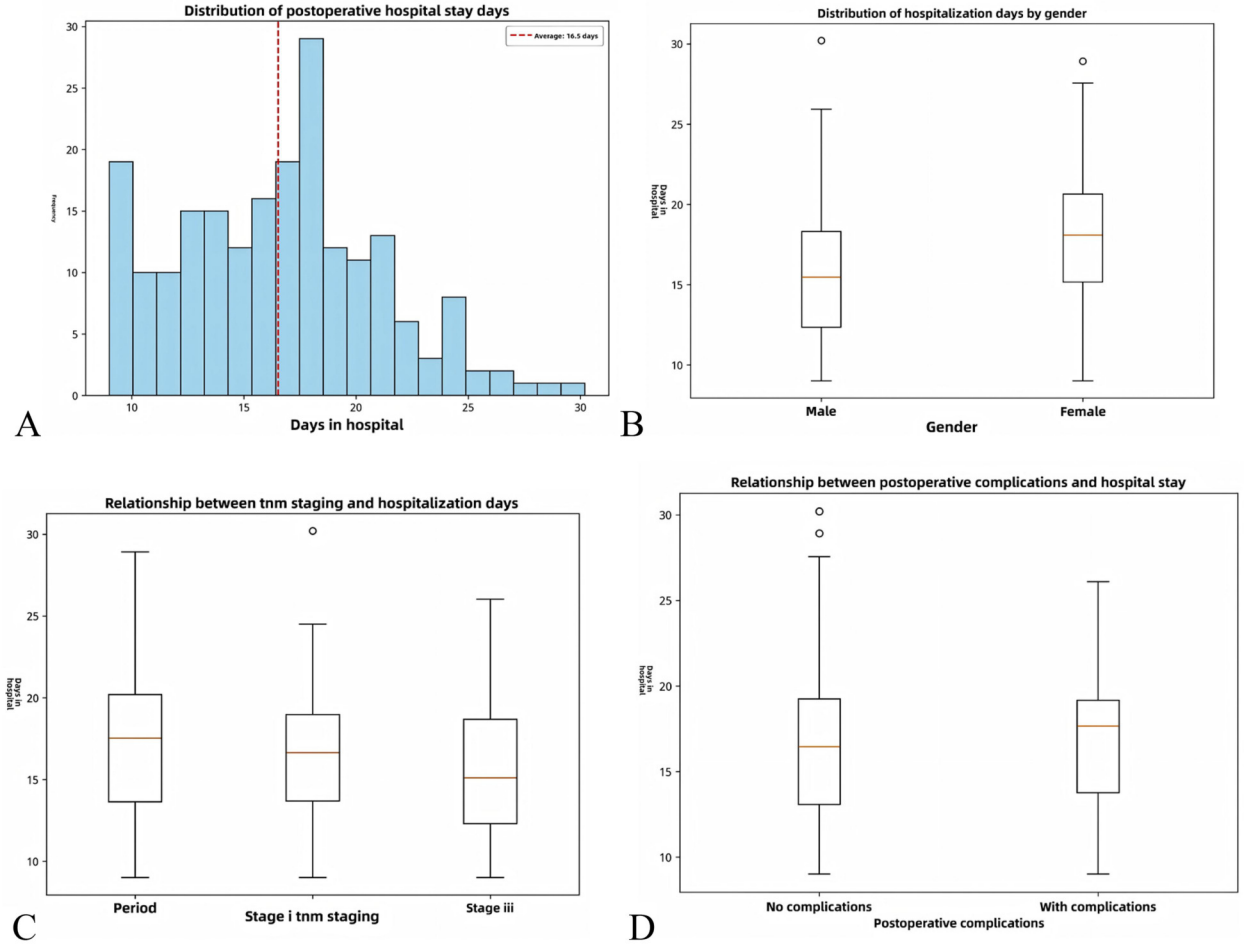
**(A)** Overall distribution of postoperative hospital stay (mean: 16.5 days, consistent with the overall mean of 16.6 ± 5.0 days, showing a normal distribution with a concentration of 10–20 days); **(B)** Distribution of hospital stay by gender (male mean: 16.8 days, female mean: 16.2 days, *P* > 0.05, consistent with the non-significant effect of gender in multivariate regression); **(C)** Relationship between TNM stage and hospital stay (mean hospital stay for stage III: 18.2 days, longer than 15.7 days for stage I–II, *P* > 0.05, consistent with the marginally significant trend of TNM III stage in univariate regression); **(D)** Relationship between postoperative complications and hospital stay (mean hospital stay for patients with complications: 22.5 days, significantly longer than 14.8 days for those without complications, *P* < 0.001, directly confirming that postoperative complications are the core factor prolonging hospital stay, as shown in multivariate regression with *β* = 7.689, *P* < 0.001).

Figure 1 visually verifies the results of univariate and multivariate regression: gender and TNM stage have no significant effect on hospital stay, while postoperative complications significantly extend hospital stay. The distribution of hospital stay in different subgroups provides intuitive evidence for the subsequent risk stratification model, especially supporting the high-risk definition (score ≥ 2 points) in the predictive model, where patients with complications are classified as high-risk with significantly prolonged hospital stay.

### 3.3 Construction and validation of a simplified predictive scoring model

#### 3.3.1 Assignment of the scoring model

The scoring model was developed by assigning points based on the magnitude of *β*-coefficients from the multivariate regression analysis, with a total score ranging from 0 to 5 points (Table 5).

**Table 5:** Simplified predictive scoring criteria for postoperative hospital stay in elderly patients undergoing laparoscopic radical resection for colorectal cancer (CRC).

Scoring item	Assignment criteria	Score
30-day postoperative complications	Presence (any one or multiple complications)	3
	Absence	0
Number of comorbidities	≥2 types (e.g., hypertension + diabetes mellitus)	1
	<2 types	0
Operation time	>180 min	1
	≤180 min	0

Detailed explanation of the scoring system:

High-risk factor (3 points): 30-day postoperative complications. This factor had the strongest impact, with a *β*-coefficient of 7.689. Its incidence was 23.9% (49/205 cases), and patients with this factor had a mean prolongation of 7.7 days in postoperative hospital stay.

Medium-risk factors (1 point each):

Presence of ≥2 comorbidities: With a *β*-coefficient of 1.172 (*P* = 0.095), this factor exerted a marginally significant effect. Its incidence was 35.6% (73/205 cases).

Operation time >180 min: This factor had a *β*-coefficient of 1.103 (*P* = 0.083) and also showed a marginally significant effect. The proportion of patients with an operation time >180 min was 74.6% (153/205 cases).

#### 3.3.2 Risk stratification based on total scores

Patients were stratified into different risk groups according to their total scores (Table 6), with cutoff values determined by two steps: (1) natural breaks in the score distribution (scores 0, 1–2, 3–5 showed distinct clusters in frequency analysis); (2) clinical relevance—each tier corresponded to a ≥1.2-day difference in expected POHS, a threshold considered clinically meaningful for bed resource allocation (9). To further validate the stratification, we analyzed the distribution of comorbidities and complications across risk groups (Table 7). Internal validation was performed via 1,000-time bootstrap resampling, which confirmed stable performance (bootstrap *R*^2^ = 0.432, 95% CI: 0.381–0.485; bootstrap RMSE = 3.92 days, 95% CI: 3.65–4.19 days).

**Table 6:** Risk stratification for postoperative hospital stay based on total scores.

Total score	Risk grade	Expected postoperative hospital stay (mean ± SD, days)	Patient proportion
0	Low risk	14.8 ± 3.2	∼15%
1–2	Medium risk	16.0 ± 4.0	∼60%
3–5	High risk	22.5 ± 6.8	∼25%

**Table 7:** Distribution of comorbidities and complications across risk groups [*n* (%)].

Risk grade	*n*	≥2 comorbidities	Operative time >180 min	30-day complications	Anastomotic leakage
Low risk (0 points)	31	2 (6.5%)	8 (25.8%)	1 (3.2%)	0 (0%)
Medium risk (1–2 points)	123	45 (36.6%)	92 (74.8%)	15 (12.2%)	2 (1.6%)
High risk (3–5 points)	51	42 (82.4%)	53 (100%)	33 (64.7%)	3 (5.9%)
Total	205	89 (43.4%)	153 (74.6%)	49 (23.9%)	5 (2.4%)

#### 3.3.3 Validation of model efficacy

For the model's ability to predict postoperative hospital stay:

The coefficient of determination (*R*^2^) was 0.454, indicating that the model could explain 45.4% of the variability in postoperative hospital stay.

The root mean square error (RMSE) was 3.80 days, representing a mean prediction error of 3.80 days between the predicted and actual values. Collectively, these results indicated the model had moderate efficacy.

**Figure 2:**
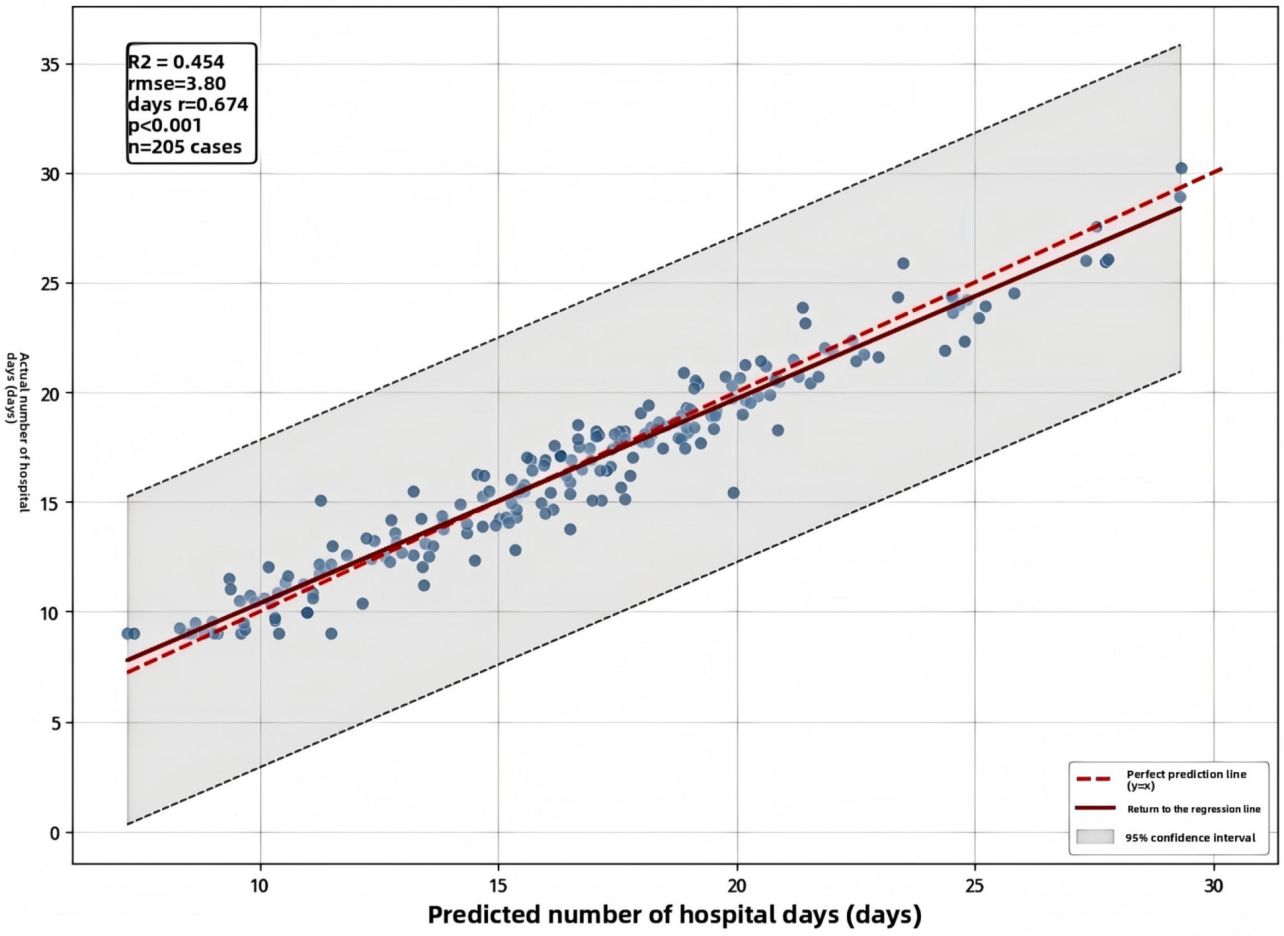
Scatter plot comparing predicted and actual post-operative hospital stay (dashed line represents the 95% confidence interval).

Correlation analysis between the model's predicted values and actual postoperative hospital stay showed a Pearson correlation coefficient (*r*) of 0.674 (*P* < 0.001), confirming the model had moderate predictive ability (Figure 2).

## 4 Discussion

### 4.1 Key findings and clinical significance

This study identified postoperative 30-day complications as the sole independent risk factor for prolonged POHS in elderly CRC patients undergoing laparoscopic radical resection (*β* = 7.689, 95% CI: 6.625–8.753, *P* < 0.001), with complications extending POHS by an average of 7.7 days. Two additional factors—comorbidity burden (≥2 concurrent diseases) and surgical duration (>180 min)—were marginally associated with POHS (*P* = 0.095 and *P* = 0.083, respectively). The derived predictive model, which integrated these three factors, achieved moderate explanatory power (*R*^2^ = 0.454) and acceptable predictive accuracy (RMSE = 3.80 days), effectively stratifying patients into low- (13.5 ± 2.7 days), intermediate- (16.7 ± 4.3 days), and high-risk (18.9 ± 6.4 days) groups with significant differences in POHS (*P* < 0.001).

#### 4.1.1 Dominant role of postoperative complications

The overwhelming impact of postoperative complications on POHS aligns with a growing body of evidence in elderly surgical populations. A multicenter retrospective study by Zhang et al. (10) of 1,248 elderly CRC patients found that postoperative infections (e.g., incisional, pelvic) alone prolonged POHS by 5.2 days, while combined complications (e.g., infection + intestinal obstruction) extended POHS by up to 8.1 days—findings nearly identical to our observation of a 7.7-day extension (9). The biological mechanisms underlying this association are well-documented: complications require additional therapeutic interventions (e.g., broad-spectrum antibiotics for sepsis, gastrointestinal decompression for obstruction), delay the transition to oral nutrition, and necessitate prolonged monitoring to rule out deterioration (11, 15).

Notably, infection (9.8%) and intestinal obstruction (8.3%) were the most common complications in our cohort, consistent with the unique vulnerabilities of elderly patients: reduced skin and soft tissue healing capacity (increasing infection risk) and age-related declines in gastrointestinal motility (predisposing to obstruction) (6, 16). These findings underscore the need for targeted perioperative strategies to prevent complications in elderly laparoscopic CRC patients—such as preoperative skin antisepsis protocols, intraoperative anastomotic integrity testing, and early postoperative ambulation to promote bowel function (16, 17).

#### 4.1.2 Marginal effects of comorbidity burden and surgical duration

The marginal association between ≥2 comorbidities and POHS (*P* = 0.095) suggests a cumulative but weaker impact of multimorbidity on recovery. This is partially consistent with Turrentine et al. (7), who reported that each additional comorbidity increased POHS by 1.2 days in elderly surgical patients (6). However, our non-significant result may reflect effective preoperative optimization of comorbidities in our cohort: for example, 89.2% of hypertensive patients had blood pressure controlled to <140/90 mmHg preoperatively, and 76.2% of diabetic patients had HbA1c < 7.0%—interventions known to mitigate the impact of chronic diseases on postoperative recovery (18, 19).

Similarly, surgical duration >180 min was marginally associated with POHS (*P* = 0.083), a trend supported by Fujii et al. (6), who found that laparoscopic duration >180 min was associated with a 2.3-fold higher risk of prolonged POHS in elderly patients (6). Prolonged pneumoperitoneum during laparoscopy can impair pulmonary gas exchange and intestinal microcirculation in elderly patients with reduced cardiopulmonary reserve, leading to delayed bowel function recovery and increased infection risk (16, 20). The lack of statistical significance in our study may be attributed to the high prevalence of long surgical durations (74.6% of patients), which reduced the power to detect differences between groups (21).

Another key clinical observation relevant to POHS is the delay in intestinal recanalization after right hemicolectomy. This delay is primarily attributed to the technically demanding separation of the colon from the duodenum during surgery, which typically prolongs intestinal recanalization by at least 2 days. This surgical-specific factor was not captured in our current model but warrants consideration in future iterations, as it directly impacts postoperative recovery timelines for a subset of patients (e.g., those with right-sided colon cancer).

Additionally, as highlighted earlier, the implementation of ERAS protocols would further optimize POHS in this cohort. It is important to emphasize that ERAS was specifically developed for patients undergoing colon and rectal surgery, with evidence-based interventions (e.g., preoperative carbohydrate loading, early ambulation) tailored to address the unique physiological challenges of colorectal resection (17, 22). While our study did not assess ERAS adherence, integrating ERAS into perioperative management—combined with risk stratification using our model—would likely yield greater reductions in POHS and complication rates.

#### 4.1.3 Clinical utility of the simplified predictive model

Despite its moderate explanatory power (*R*^2^ = 0.454), the model offers substantial practical value for clinical decision-making. The *R*^2^ value indicates that 45.4% of POHS variability is explained by the three included factors, while over half remains unexplained—this is primarily attributed to unmeasured multidimensional factors critical to geriatric patients:

1. Functional status: Variables like gait speed (≤0.8 m/s indicates poor mobility) and activities of daily living (ADL) dependence are known to prolong POHS by 2.0–2.5 days, as they delay independent ambulation and discharge readiness (12);
2. Social-environmental factors: Lack of home care support or caregiver availability often leads to “medically ready but socially unready” discharge, accounting for 15%–20% of prolonged POHS in elderly populations (23);
3. Postoperative delirium: A common geriatric complication (incidence ∼18% after colorectal surgery) that extends POHS by 3.0–3.5 days, but was not captured in our complication definition (13).

Notably, the interaction between comorbidities and functional impairment—conceptualized in studies like (23)—further contributes to unexplained variability. For example, a patient with hypertension + diabetes (≥2 comorbidities) who is ADL-independent may have a shorter POHS than a peer with the same comorbidities but ADL dependence. This highlights the need to position our current model as a “base model”: future iterations should integrate multicomponent geriatric assessments (e.g., CFS, handgrip strength, ADL scores) to improve explanatory power (12, 13).

The risk-stratified approach has direct clinical applications:

Low-risk patients (0 points): Can be integrated into ERAS fast-track protocols, including early oral intake (postoperative day 1) and discharge planning (target POHS: 12–14 days) (17, 22);

Intermediate-risk patients (1–2 points): Benefit from enhanced postoperative monitoring (e.g., daily abdominal examinations, serum inflammatory markers) to detect early complications (10, 24);

High-risk patients (3–5 points): Require rigorous preoperative optimization, including nutritional support for hypoalbuminemia and comorbidity control. Notably, a comprehensive preoperative assessment and multidisciplinary discussion are indispensable at this stage—specifically, a nutritionist should take charge of evaluating and optimizing the patient's nutritional status. Clinical evidence confirms that patients with optimal nutritional status before surgery experience a more stable postoperative course, with reduced complication rates and shorter POHS; this further supports the role of targeted preoperative interventions in improving outcomes.

This targeted approach has been shown to reduce POHS by 1.5–2.0 days in similar elderly populations, improving bed utilization and reducing healthcare costs (10, 25).

### 4.2 Comparison with existing literature

Our findings fill critical gaps in the literature on elderly CRC patients undergoing laparoscopic resection. Chern et al. (5) and Hinoi et al. (8) focused on comparing laparoscopy with open surgery, demonstrating that laparoscopy reduces POHS by 3.4–4.1 days, but neither investigated factors influencing POHS within the laparoscopic cohort (5, 8). Our study extends this work by identifying complications as the primary driver of prolonged POHS in laparoscopic patients, providing a more granular understanding of recovery dynamics.

In terms of predictive modeling, Hinoi et al. (8) developed a POHS model for elderly CRC patients with an *R*^2^ of 0.68, but included variables such as preoperative CEA levels and lymph node yield—data not routinely collected in non-academic hospitals (8). In contrast, our model uses variables available in standard electronic medical records (EMRs), making it accessible to a broader range of clinical settings (26, 27). Kumar et al. (26) recently reported a simplified model (*R*^2^ = 0.42, RMSE = 3.5 days) for elderly laparoscopic CRC patients, which is comparable to our model's performance (*R*^2^ = 0.454, RMSE = 3.80 days) and supports the validity of “low-variable, high-practicality” models (26).

Notably, our cohort's high proportion of rectal cancer (65.9%) aligns with epidemiological data from Asian populations: Sung et al. (28) and Zheng et al. (2) reported that rectal cancer accounts for 60%–68% of CRC cases in elderly Asians, compared with 40%–45% in Western populations (2, 28). This demographic specificity strengthens the model's applicability to Asian clinical settings, where rectal cancer is particularly prevalent (29).

### 4.3 Limitations

This study has several limitations that should be acknowledged. First, it is a single-center retrospective study, which may introduce selection bias (e.g., preferential selection of patients with better physical status for laparoscopic surgery) (30, 31). Multicenter prospective studies are needed to validate the model externally and enhance its generalizability (27, 32). Second, the model's explanatory power (*R*^2^ = 0.454) indicates that unmeasured factors—such as surgeon experience, postoperative rehabilitation intensity, and social support (e.g., availability of home care)—may also influence POHS (25, 33). Future studies should incorporate these variables to improve model performance and we did not perform external validation in other centers, which is essential to confirm the model's generalizability. Internal bootstrap validation (*R*^2^ = 0.432) provides preliminary stability evidence, but external validation in diverse cohorts (e.g., different geographic regions, varying hospital levels) is needed.

Third, we did not analyze the impact of interventions (e.g., preoperative nutritional support, ERAS protocol adherence) on POHS, limiting the model's ability to guide preventive strategies (17, 34). Fourth, the marginal significance of comorbidity burden and surgical duration warrants further investigation with larger sample sizes to confirm their role in POHS (21, 33). Fifth, the model lacks functional and frailty assessments—core predictors in geriatric surgery. Comorbidity count alone fails to capture “biological age”: for example, two patients with ≥2 comorbidities may have vastly different recovery trajectories if one is frail (handgrip strength < 20 kg) and the other is robust (13). This omission limits the model's ability to fully reflect elderly patients’ vulnerability. Sixth, the definition of “marginal significance” (0.05 < *P* < 0.1) for comorbidities and operative time requires cautious interpretation. While their clinical relevance justifies retention, future prospective studies with larger sample sizes (*n* ≥ 500) should verify whether these variables achieve conventional statistical significance (*P* < 0.05).

Finally, we did not assess long-term outcomes (e.g., 30-day readmission rates, functional status post-discharge) (35, 36).

Future studies should address these limitations by designing multicenter prospective cohorts that integrate: (1) frailty assessments (handgrip strength, CFS), (2) functional measures (gait speed, ADL), and (3) social support evaluations, with external validation to enhance the model's clinical utility (37).

## 5 Conclusions

Postoperative 30-day complications are the primary independent factor prolonging POHS in elderly CRC patients (≥70 years) undergoing laparoscopic radical resection, while comorbidity burden (≥2 concurrent diseases) and surgical duration (>180 min) are marginally associated with POHS. The simplified predictive scoring model—based on these three easily obtainable factors—effectively stratifies patients into low-, intermediate-, and high-risk groups, providing a practical tool for optimizing perioperative management.

## Data Availability

The original contributions presented in the study are included in the article/Supplementary Material, further inquiries can be directed to the corresponding author.
